# Substrate Activation of the Low-Molecular Weight Protein Tyrosine Phosphatase from *Mycobacterium tuberculosis*

**DOI:** 10.1021/acs.biochem.0c00059

**Published:** 2020-03-06

**Authors:** Alessandra Stefan, Fabrizio Dal Piaz, Antonio Girella, Alejandro Hochkoeppler

**Affiliations:** † Department of Pharmacy and Biotechnology, University of Bologna, 40136 Bologna, Italy; ‡ CSGI, University of Firenze, 50019 Sesto Fiorentino, Firenze, Italy; § Department of Medicine, University of Salerno, 84084 Fisciano, Salerno, Italy

## Abstract

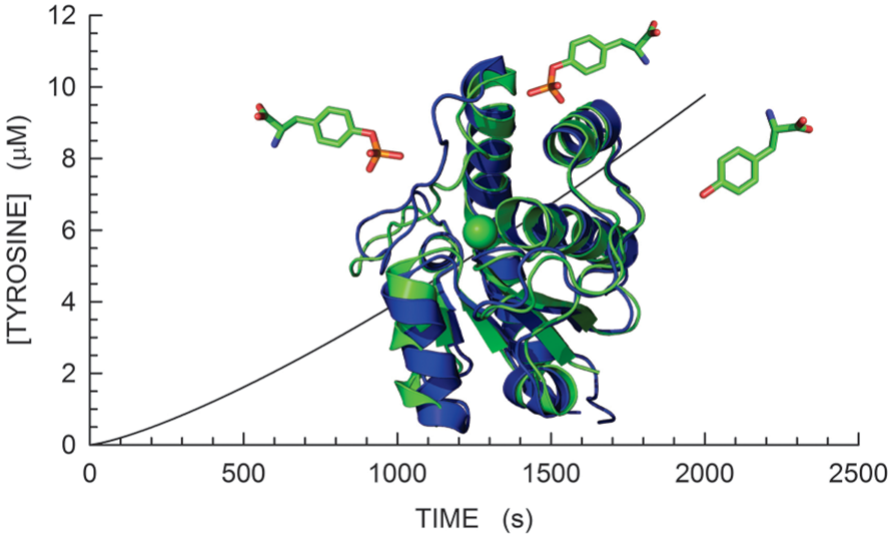

*Mycobacterium tuberculosis* is known to express a low-molecular weight protein tyrosine phosphatase. This enzyme, denoted as MptpA (*Mycobacterium* protein tyrosine phosphatase A), is essential for the pathogen to escape the host immune system and therefore represents a target for the search of antituberculosis drugs. MptpA was shown to undergo a conformational transition during catalysis, leading to the closure of the active site, which is by this means poised to the chemical step of dephosphorylation. Here we show that MptpA is subjected to substrate activation, triggered by *p*-nitrophenyl phosphate or by phosphotyrosine. Moreover, we show that the enzyme is also activated by phosphoserine, with serine being ineffective in enhancing MptpA activity. In addition, we performed assays under pre-steady-state conditions, and we show here that substrate activation is kinetically coupled to the closure of the active site. Surprisingly, when phosphotyrosine was used as a substrate, MptpA did not obey Michealis–Menten kinetics, but we observed a sigmoidal dependence of the reaction velocity on substrate concentration, suggesting the presence of an allosteric activating site in MptpA. This site could represent a promising target for the screening of MptpA inhibitors exerting their action independently of the binding to the enzyme active site.

Protein functions can be controlled in a number of ways, among which the competing actions of protein kinases and phosphatases are of great relevance, due to their rapid execution and to their usefulness for the amplification of biochemical signals.^1,2^ The main targets of protein phosphorylation by kinases are serine, threonine, and tyrosine,^3^ whose dephosphorylation can be catalyzed by phosphatases featuring different specificity. When phosphotyrosine (pTyr) is considered, two functional types of phosphatases are known, i.e., those strictly specific for pTyr, and enzymes exerting a dual action, e.g., toward both phosphothreonine and phosphotyrosine.^4^ In addition, pTyr-specific phosphatases are distinguished according to their molecular mass, and they are conventionally classified as high-molecular weight (HMW, >20 kDa) and low-molecular weight (LMW, ≤20 kDa) enzymes. The importance of pTyr-specific phosphatases (PTPases) in the onset of human infectious diseases is well-known.^5−8^ In particular, the *Mycobacterium tuberculosis* MptpA enzyme is a LMW PTPase (17.9 kDa) that can dephosphorylate the host VPS33B (vacuolar protein sorting 33B) protein. This dephosphorylation inactivates VPS33B,^9^ inhibits phagosome–lysosome fusion,^10,11^ and therefore confers virulence to the pathogen.^12^ Accordingly, MptpA represents an interesting target for the search of antituberculosis drugs, and quite a number of potential MptpA inhibitors have been reported.^13−15^ Moreover, to facilitate the identification of an effective and selective MptpA inhibitor, the repertoire of MptpA substrates is currently being investigated.^16^

The catalytic mechanism of PTPases was mainly investigated using three enzymes as model systems, i.e., the human placental protein tyrosine phosphatase 1B (PTP1B), its rat homologue PTP1, and the *Yersinia* sp. Yop51 enzyme.^17−21^ Site-specific variants of the *Yersinia* sp. and of the PTP1 enzyme were used to show that the thiolate group of a special cysteine featuring an unusually low p*K*_a_^20^ is essential for the nucleophilic attack to the phosphate group bound to tyrosine.^21,22^ This nucleophilic attack is assisted by an essential arginine, as revealed by the R409A variant of the *Yersinia* sp. enzyme,^23^ and by structural studies suggesting that the guanidinium group of R409 interacts with two oxygens of the pTyr phosphate group.^24^ Finally, it was noted that the cysteine and the arginine essential for catalysis are conserved in all PTPases, and that they are spaced by five amino acids, whose identity can diverge. Accordingly, the C(XXXXX)R motif was recognized as the signature for both prokaryotic and eukaryotic PTPases and for both HMW and LMW forms. In addition, the catalytic cycle of PTPases was shown to require an amino acid acting as an acid first and subsequently as a base. In the *Yersinia* sp. PTPase, this requirement is fulfilled by an aspartic acid (D356),^25^ which transfers a proton to the leaving tyrosine, and subsequently, the conjugated aspartate activates a water molecule, generating the secondary nucleophile responsible for the attack to the phosphoenzyme. Interestingly, the essential cysteine, arginine, and aspartate of PTPases are located in loops connecting elements of the secondary structure. The loop connecting cysteine and arginine is conventionally denoted as the phosphate loop (P-loop), and the stretch of amino acids containing the catalytic aspartate is denominated the D-loop.

The strict specificity of some PTPases toward pTyr depends on a hydrophobic pocket shaped to position the phosphate of pTyr in line with the nucleophilic cysteine,^24,26^ and inducing a very unfavorable geometry for the nucleophilic attack when the phosphate is bound to a shorter amino acid, i.e., serine or threonine.^27^ Once pTyr is bound by PTPases, a large conformational rearrangement occurs at the expense of the D-loop. It was indeed shown that this loop moves toward the P-loop by several angstroms, converting the enzyme conformation from an open to a closed, catalytically competent, form.^24^ The understanding of the strict specificity for pTyr and of the interconversion of open to closed enzyme forms during catalysis, prompted the search for specific inhibitors targeting the active site. However, the high degree of conservation of the mechanism underlying pTyr dephosphorylation hampered the identification of highly selective inhibitors. Nevertheless, the identification of a secondary substrate binding site in PTPases,^28^ or noncatalytic phosphoryl binding site, triggered the search for allosteric inhibitors.^29,30^ These inhibitors might indeed feature high selectivity, arising from the structural divergence of the enzyme regions shaping the allosteric site. Remarkably, one allosteric inhibitor was shown to behave noncompetitively and was able to trap PTP1B in the open conformation,^30^ the competence of which in catalysis is drastically reduced. It is important to note that the binding of this allosteric inhibitor^30^ occurs at a site that is different from that previously found to bind a second substrate molecule.^28^

In addition to their competence in binding inhibitors, allosteric sites in PTPases can be also supposed to represent targets for enzyme activation, mediating conformational rearrangements favorable to catalysis. However, no information about this issue is yet available for PTPases. Therefore, we thought it of interest to investigate this point, and we decided to use as a model system the LMW tyrosine phosphatase from *M. tuberculosis* (MptpA), the open and closed structures of which are known,^31,32^ and whose essential residues for catalysis were identified (Figure 1^33^). The kinetics of the hydrolysis of *p*-nitrophenyl phosphate (*p*-NPP) and pTyr catalyzed by MptpA is presented here, along with the characterization of enzyme activation induced by both substrates. In addition, the kinetic coupling of substrate activation to the closure of enzyme active site is also reported.

**Figure 1:**
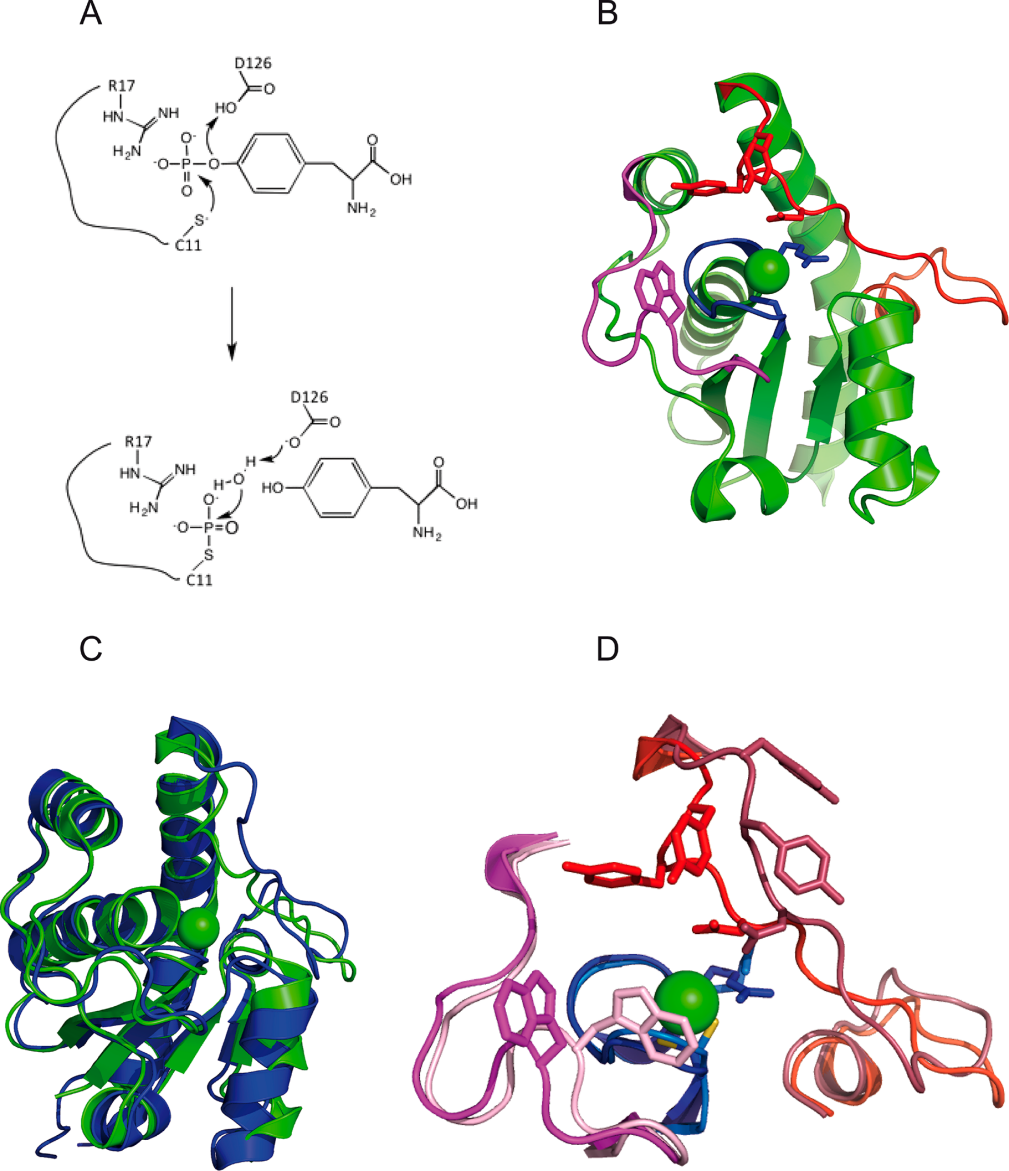
Catalytic mechanism and tertiary structure of MptpA. (A) Active site of MptpA, with the primary and secondary nucleophilic attack exerted by enzyme cysteine 11 and by an activated H_2_O molecule, respectively. (B) Tertiary structure of the closed conformation of MptpA (Protein Data Bank entry 1u2p). The P-loop, W-loop, and D-loop are colored blue, magenta, and red, respectively. The green sphere represents a chloride ion. (C) Superposition of the closed and open conformations of MptpA (Protein Data Bank entries 1u2p and 2luo), colored green and blue, respectively. (D) Detail of the superposition shown in panel C. The P-loop, W-loop, and D-loop of the closed conformation are colored blue, magenta, and red, respectively. The corresponding loops of the open conformation are colored cyan, pink, and salmon, respectively. The tertiary structure representations were rendered with PyMol (The PyMOL Molecular Graphics System, version 1.3, Schrödinger, LLC).

## Materials and Methods

### Bacterial Strains, Plasmids, and Media

*Escherichia coli* BL21(DE3) [F-, *dcm, ompT, hsdS(r*_*B*_^*–*^*m*_*B*_^*–*^), *gal*, λ(DE3)] was obtained from Novagen (Madison, WI) and used for protein expression. An artificial gene encoding MptpA (UniProt entry P9WIA1), and optimized for *E. coli* codon usage, was synthesized (GenScript, Piscataway, NJ). The synthetic gene was cloned into the pETDuet-1 plasmid using the *Nco*I and *Pst*I sites, yielding the recombinant pETDuet1-MptpA construct. To keep in frame the CDS of MptpA, an additional codon for glycine (GGA) was inserted into the synthetic gene after the start codon located in the *Nco*I palindrome [CCATGG (Figure S1)]. BL21(DE3) competent cells were transformed by electroporation (Gene Pulser II, Bio-Rad, Hercules, CA) with 5 ng of the recombinant construct, and transformants were selected on LB agar Petri dishes (tryptone, yeast extract, NaCl, and agar at 10, 5, 10, and 15 g/L, respectively) containing ampicillin (100 μg/mL). Bacterial cultures were grown (at 37, 30, or 15 °C) under shaking conditions (180 rpm) using LB medium supplemented with ampicillin. The expression of MptpA was induced by the addition of 1 mM isopropyl β-d-1-thiogalactopyranoside (IPTG) to the culture medium.

### Protein Overexpression

Single colonies of transformants were grown in LB-ampicillin medium for 15 h at 37 °C. These precultures were then diluted (1:500) into fresh medium (250 mL) and grown at 30 °C until the population density corresponded to an OD_600_ of ∼1.2. The inducer (IPTG) was then added, and the cultures were grown at 15 °C for 16 h. Cells were harvested by centrifugation (4500*g* for 20 min), and the pellets were resuspended in 25 mL of 50 mM Tris-HCl (pH 8.0), 150 mM NaCl, 1 mM EDTA, and 1 mM phenylmethanesulfonyl fluoride (PMSF). Total protein extracts were obtained by sonication (Misonix-3000 sonifier, output level of 18 W for 15 s, followed by a 15 s cooling interval, for four cycles), and the soluble fraction was isolated by centrifugation (12000*g* for 30 min). The protein concentration was determined according to Bradford.^34^

### MptpA Purification

#### Ammonium Sulfate Precipitation

To the soluble fraction, chilled on ice, was slowly added solid ammonium sulfate (a.s.) under constant stirring to reach 20% a.s. saturation. After equilibration for 40 min, the sample was centrifuged at 12000*g* for 30 min at 4 °C and the supernatant was recovered. The concentration of ammonium sulfate in the sample was slowly increased to 60% saturation, and the mixture was stirred for 40 min on ice. After centrifugation at 12000*g* for 30 min at 4 °C, the supernatant was discarded and the protein pellet was resuspended in 20 mL of buffer A [50 mM Tris-HCl (pH 7.5), 3 M NaCl, and 1 mM EDTA].

#### HiScreen Phenyl Sepharose FF Chromatography

The resuspended protein pellet was loaded (flow rate of 1 mL/min) onto a 5 mL HiScreen Phenyl Sepharose FF column (GE Healthcare, Piscataway, NJ) previously equilibrated with buffer A. The column was then washed with 10–15 column volumes of equilibration buffer. The elution was performed with a linear 3 to 0 M NaCl gradient (10 column volumes, flow rate of 1 mL/min), and fractions of 1 mL were collected. A final elution step with water was also performed. Eluted fractions were analyzed by sodium dodecyl sulfate–polyacrylamide gel electrophoresis (SDS–PAGE) (15% acrylamide).

#### Gel Filtration Chromatography

The best fractions from the HiScreen Phenyl Sepharose column containing MptpA were pooled and concentrated by ultrafiltration with an Amicon stirred cell (Merck Millipore, Darmstadt, Germany) equipped with a YM10 membrane. The concentrated sample (2 mL) was loaded onto a Superdex-200 column (GE Healthcare, 1.6 cm × 62 cm), equilibrated with 50 mM Tris-HCl (pH 8.0), 150 mM NaCl, and 1 mM EDTA (flow rate of 0.6 mL/min), and 0.9 mL fractions were collected. The best eluted fractions, according to SDS–PAGE, were then pooled, concentrated, and stored at −20 °C.

### Phosphatase Activity Assays

Enzyme assays were performed using a Cary 300 ultraviolet–visible spectrophotometer in the presence of *p-*NPP (Sigma-Aldrich, Millipore Sigma, Darmstadt, Germany) or phosphotyrosine (pTyr) (Sigma-Aldrich) as the substrate. The activity assay buffer contained 50 mM Tris-HCl (pH 8.0), 2 mM EDTA, and (unless otherwise stated) 420 nM enzyme, in a final volume of 1 mL. To calculate the catalytic constants of MptpA under different conditions, the Levenberg–Marquardt algorithm in SigmaPlot 14 (Systat Software, San Jose, CA) was used. When *p-*NPP was used as the substrate, the absorbance changes at 405 nm were recorded and the molar extinction coefficient (ε) for *p*-nitrophenolate was assumed to be equal to 18.3 mM^–1^ cm^–1^.^35^ When the initial velocity of reactions at the expense of pTyr was assayed, Absorbance changes were determined at 282 nm, and the difference in extinction between tyrosine and phosphotyrosine (Δε_Tyr-pTyr_) was determined to be equal to 0.96 mM^–1^ cm^–1^.

### Kinetics of Substrate Activation

The observed kinetics detected in the presence of low concentrations (e.g., 0.5 mM) of pTyr or *p*-NPP were interpreted according to the following model of substrate activation:
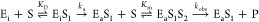
1where E_i_ is the free inactive enzyme, *K*_D_ is the dissociation constant of the E_i_S_1_ complex, E_a_ indicates the activated enzyme, and *k*_a_ is the activation rate constant. It should be noted that *k*_a_ is interpreted as a net activation rate constant.^36^ The conversion of the inactive to active enzyme is a reversible process:
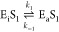
2

Accordingly, the net activation rate constant^36^ (*k*_a_) is defined as the true forward rate constant (*k*_1_) multiplied by the proportion of enzyme channeled toward product formation from the step considered irreversible and can be calculated via
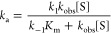
3At low substrate concentrations (≪*K*_m_), and assuming that the first molecule of the substrate is bound to the allosteric (activation) site of the enzyme and that this substrate molecule does not undergo dephosphorylation, the concentration of the E_i_S_1_ complex can be calculated as
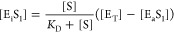
4where [E_T_] indicates the total enzyme concentration. The concentration of the E_a_S_1_ complex can accordingly be determined:
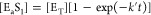
5where *k*′ indicates *k*_a_[S]/(*K*_D_ + [S]) and *t* indicates time.

Finally, the initial velocity is defined as
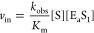
6and, considering [S] as constant ([S] ≫ [E_T_]), the generation of product as a function of time can be estimated:
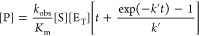
7

### Effect of Phosphoserine on MptpA Activity

To test the effect, if any, of *O*-phospho-l-serine (pSer) (Sigma-Aldrich) on phosphatase activity, the reaction velocity was assayed in a final volume of 1 mL using a constant concentration of 1 or 10 mM pSer and varying the *p*-NPP concentration (from 0.5 to 25 mM). Similar assays were performed by adding 10 mM serine (Ser) to the enzyme/substrate mixture.

### Effect of Phosphate on MptpA Activity

To assay the inhibition exerted by orthophosphate on phosphatase activity, the release of *p*-nitrophenol was monitored at 405 nm, in the presence of different concentrations of *p*-NPP (0.5–25 mM), and in the presence of a constant concentration of phosphate (1 or 10 mM).

### Stopped-Flow Analyses

The hydrolysis of *p*-NPP catalyzed by MptpA W152F under pre-steady-state conditions was analyzed using a KinTek SF2004 stopped-flow instrument (KinTek, Snow Shoe, PA). To determine tryptophan fluorescence, samples were excited at 280 nm and the emission was detected using a long-pass filter. The release of *p*-nitrophenolate was estimated by monitoring the absorbance changes at 405 nm. All reactions were assayed at 20 °C. The enzyme syringe contained MptpA W152F [4.5 μM, in Tris-HCl (pH 8.0) and 2 mM EDTA], and the substrate syringe contained 1 mM *p*-NPP in the same buffer. Usually, 20 traces were averaged.

### Surface Plasmon Resonance Analyses

The binding of pTyr to MptpA W152F was analyzed using a Biacore 3000 instrument (GE Healthcare) according to previously published procedures.^37^ MptpA W152F surfaces were prepared on research grade CM5 sensor chips (GE Healthcare) by immobilizing the protein [100 μg/mL in 10 mM CH_3_COONa (pH 5.0)] using a standard amine-coupling protocol; this procedure led to an observed density of 3.5 kRU. The substrate pTyr was dissolved and diluted in HPS-EP buffer [0.01 M HEPES (pH 7.4), 0.15 M NaCl, 3 mM EDTA, and 0.005% (v/v) surfactant P20] to obtain samples at five different concentrations (25, 65, 150, 400, and 1000 nM).

Binding experiments were performed at 25 °C, using a flow rate of 50 mL/min, with 20 s of association time and 100 s of dissociation time. Resulting curves were fitted to single-site (1:1 binding) and double-site (heterogeneous ligand) interaction models, yielding a single *K*_D_ and two *K*_D_’s, respectively. Sensorgram elaboration was performed using the BIAevaluation software, provided by GE Healthcare.

## Results and Discussion

**Figure 2:**
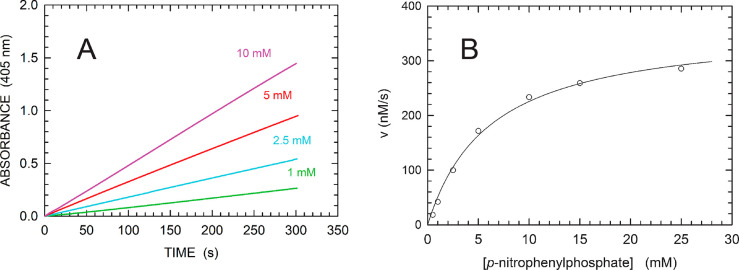
Kinetics of *p*-NPP hydrolysis catalyzed by MptpA. (A) Time course of reactions monitored at 405 nm in the presence of 420 nM enzyme and *p*-NPP at the indicated concentrations. (B) Dependence of initial reaction velocities on *p*-NPP concentration. The solid line represents the best fit of the Michaelis–Menten equation to the experimental observations.

To overexpress MptpA in *E. coli*, a synthetic gene encoding *M. tuberculosis* protein tyrosine phosphatase was cloned into expression vector pETDuet-1, yielding pETDuet1-MptpA. The overexpression of this synthetic gene, optimized for *E. coli* codon usage (Figure S1), was detected at high levels in *E. coli* strain BL21(DE3), from which the target protein was mainly recovered in the soluble fraction (Figure S2). In addition, using standard chromatographic procedures we were able to isolate homogeneous MptpA (Figure S2), with a purification yield equal to 15 mg of purified enzyme from 1 L of bacterial culture. To test the catalytic performances of purified MptpA, we first assayed the phosphatase activity exerted by the enzyme at the expense of *p*-NPP. In the presence of this substrate, Michaelis–Menten kinetics was observed, and *V*_max_, *k*_cat_, and *K*_m_ were determined to be equal to 364 ± 15 nM/s, 0.87 ± 0.04 s^–1^, and 6.14 ± 0.71 mM, respectively (Figure 2 and Table 1). These values are in agreement with the *K*_m_ previously reported by Madhurantakam et al. (2.86 ± 0.03 mM^31^) and with the *k*_cat_ of ∼1 s^–1^ that can be estimated from the kinetic characterization of MptpA performed by Stehle et al. (Figure S2 in ref (32)).

**Table 1:** Values of *K*_m_, *V*_max_, and *k*_cat_ Determined for MptpA Using *p*-NPP or pTyr as the Substrate^a^

	*K*_m_ (mM)	*V*_max_ (nM/s)	*k*_cat_ (s^–1^)
*p*-NPP	6.1 ± 0.7	364 ± 15	0.87 ± 0.04
*p*-NPP	7.0 ± 1.1	360 ± 23	0.86 ± 0.05
*p*-NPP and 1 mM pSer	5.6 ± 0.5	400 ± 12	0.95 ± 0.03
*p*-NPP and 10 mM pSer	4.6 ± 0.9	612 ± 40	1.46 ± 0.10
*p*-NPP	9.4 ± 1.2	346 ± 20	0.82 ± 0.05
*p*-NPP and 10 mM Ser	8.9 ± 0.5	311 ± 7	0.74 ± 0.02
*p*-NPP	6.2 ± 1.0	486 ± 29	1.16 ± 0.07
*p*-NPP and 1 mM P_i_	5.9 ± 0.5	450 ± 15	1.07 ± 0.04
*p*-NPP and 10 mM P_i_	16.8 ± 1.4	421 ± 19	1.00 ± 0.05
pTyr	5.4 ± 0.2	288 ± 10	0.69 ± 0.02

a The effect of phosphoserine, serine, or orthophosphate (at the indicated concentrations) on the catalytic constants is also reported.

**Figure 3:**
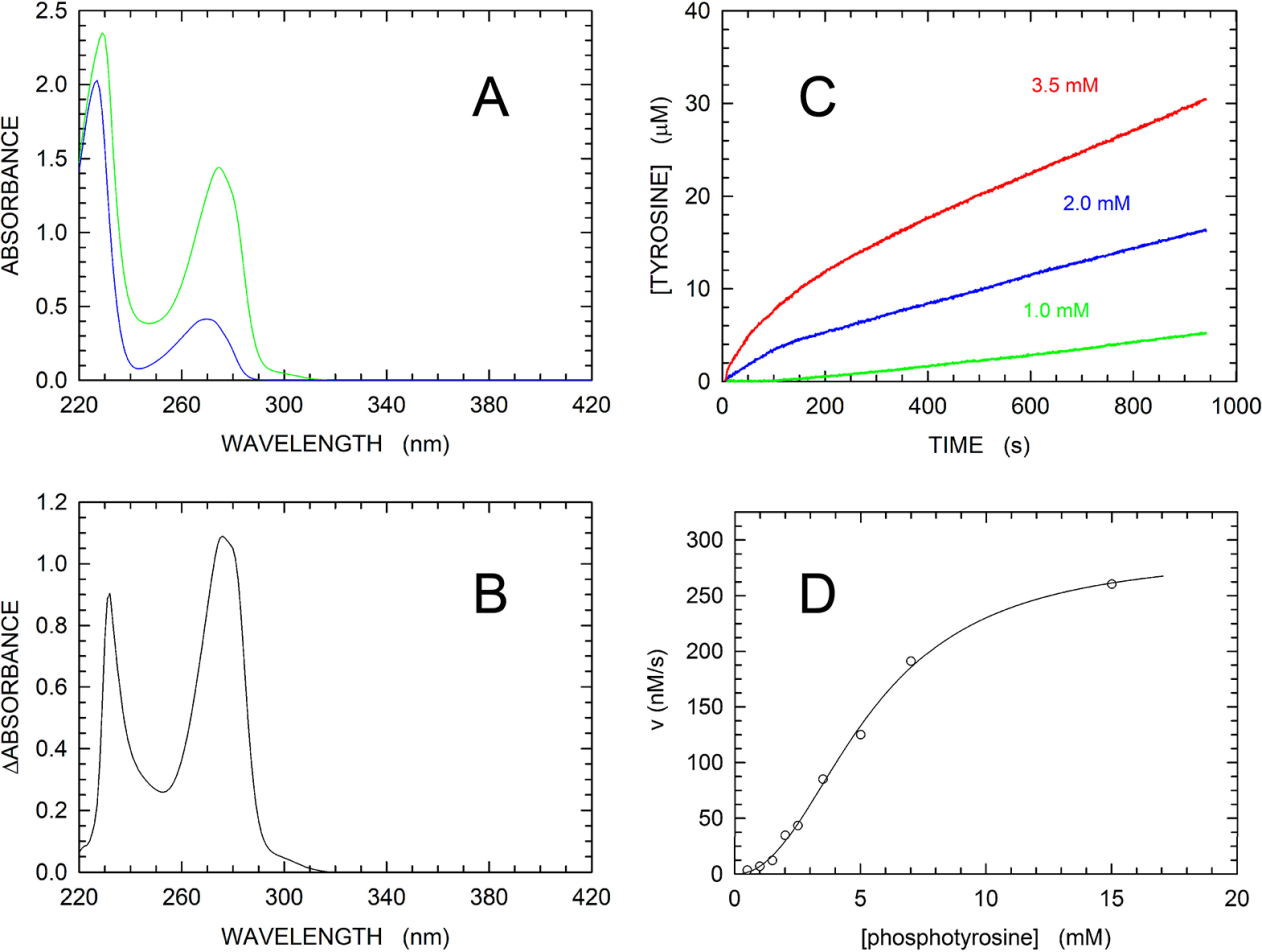
Absorption spectra of tyrosine and phosphotyrosine. Kinetics of pTyr hydrolysis catalyzed by MptpA. (A) Absorption spectra of solutions containing 1 mM tyrosine (green) and 1 mM phosphotyrosine (blue). (B) Difference absorption spectrum (tyrosine minus phosphotyrosine) of the spectra shown in panel A. (C) Time course of reactions monitored at 282 nm in the presence of 420 nM enzyme and 1, 2, or 3.5 mM pTyr (green, blue, and red, respectively). (D) Dependence of initial reaction velocity on pTyr concentration. The solid line represents the best fit of the Hill equation to the experimental observations.

To perform further activity assays using pTyr as the substrate, we compared the absorbance spectra of equimolar (1 mM) solutions of tyrosine and phosphotyrosine, at pH 8 (50 mM Tris-HCl). A higher absorbance was observed in the presence of tyrosine over the wavelength interval from 220 to 320 nm (Figure 3A). In addition, when the difference spectrum was calculated, we determined a difference extinction coefficient equal to 0.96 mM^–1^ cm^–1^ at 282 nm (Figure 3B). It should be noted that the maximal difference in absorption between tyrosine and phosphotyrosine was detected at 276 nm [where Δε_Tyr-pTyr_ = 1.09 mM^–1^ cm^–1^ (Figure 3B)]. However, to contain the absorption of the substrate, we decided to assay the dephosphorylation of pTyr at 282 nm. Surprisingly, in the presence of low concentrations of pTyr (e.g., 1 mM), a pronounced lag phase was observed before steady-state kinetics was attained (Figure 3C), suggesting the occurrence of substrate activation in *M. tuberculosis* MptpA. When higher concentrations of pTyr (e.g., 2 or 3.5 mM) were used in the assays, an initial fast reaction phase was observed, followed by a slower phase taking place in approximately 300–400 s (Figure 3C). In addition, the dependence of reaction velocity on substrate concentration did not obey Michaelis–Menten kinetics (Figure 3D). Indeed, the occurrence of a pronounced sigmoidal dependence was observed, suggesting that the enzyme features allosteric transitions when exposed to pTyr (Figure 3D). Accordingly, by fitting the Hill equation to the experimental observations, we determined *V*_max_, *k*_cat_, *K*_m_, and the Hill coefficient to be equal to 288 ± 10 nM/s, 0.69 ± 0.02 s^–1^, 5.36 ± 0.25 mM, and 2.21 ± 0.14, respectively (Figure 3D and Table 1).

**Figure 4:**
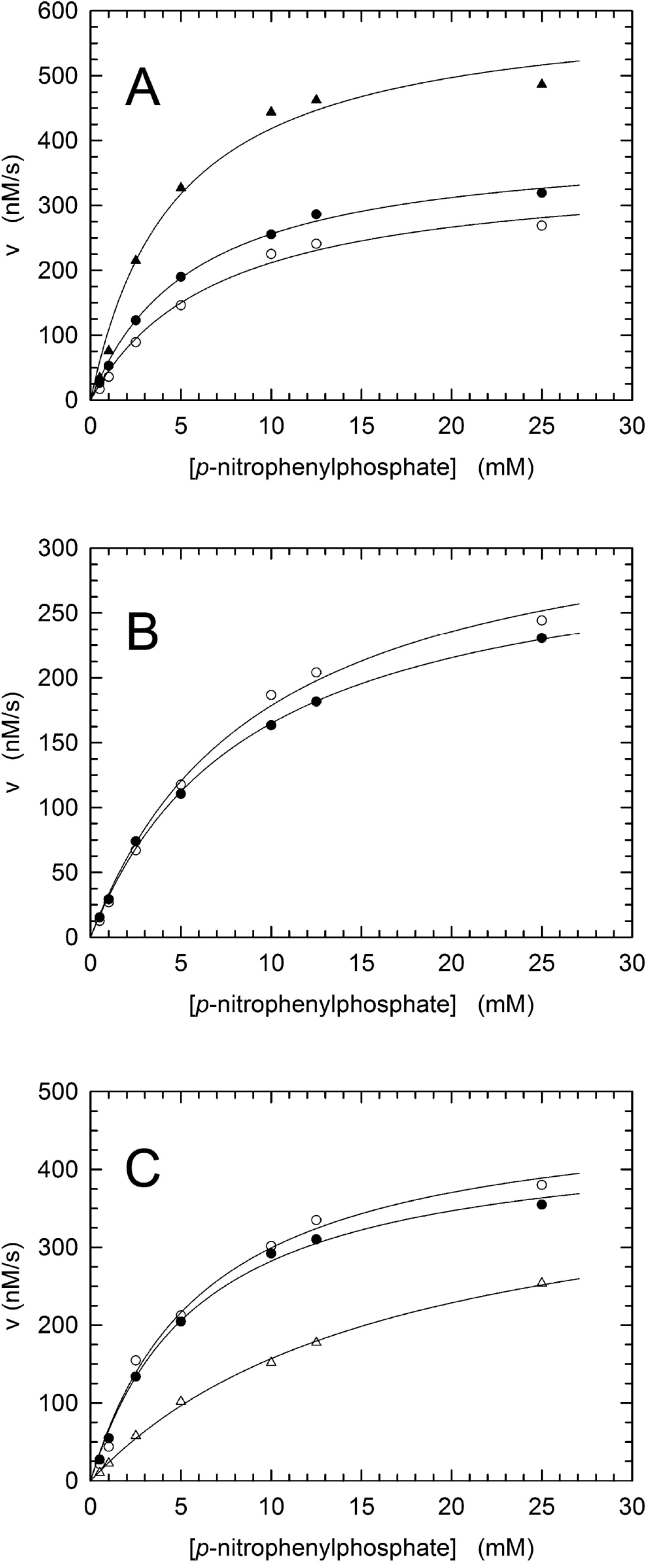
Activation and inhibition of MptpA. (A) Initial velocities of *p*-NPP hydrolysis as a function of substrate concentration, assayed in the absence (○) or presence of 1 mM (●) or 10 mM (▲) phosphoserine. (B) Initial velocities of *p*-NPP hydrolysis as a function of substrate concentration, assayed in the absence (○) or presence of 10 mM (●) serine. (C) Initial velocities of *p*-NPP hydrolysis as a function of substrate concentration, assayed in the absence (○) or presence of 1 mM (●) or 10 mM (△) orthophosphate.

To ascertain the presence of an allosteric site in MptpA, we assayed the enzyme activity at the expense of *p*-NPP in the absence or presence of phosphoserine (pSer). When initial reaction velocities were observed as a function of *p*-NPP concentration in the absence of pSer, *V*_max_, *k*_cat_, and *K*_m_ were determined to be equal to 360 ± 23 nM/s, 0.86 ± 0.05 s^–1^, and 6.99 ± 1.12 mM, respectively (Figure 4A and Table 1). These values were not significantly affected by the addition of 1 mM pSer to reaction mixtures, being equal to 400 ± 12 nM/s, 0.95 ± 0.03 s^–1^, and 5.63 ± 0.48 mM, respectively (Figure 4A and Table 1). However, a higher *V*_max_ and a higher *k*_cat_ were observed in the presence of 10 mM pSer, i.e., 612 ± 40 nM/s and 1.46 ± 0.07 s^–1^, respectively, with *K*_m_ not being affected by 10 mM pSer, i.e., 4.64 ± 0.90 mM (Figure 4A and Table 1). Remarkably, 10 mM serine did not alter either the *V*_max_ or the *K*_m_ of MptpA (Figure 4B and Table 1), suggesting that the activation of MptpA requires the binding to the allosteric site of a phosphorylated compound.

When possible mechanisms of inhibition of PTPases are considered, orthophosphate can be supposed to bind to the active site, acting as a competitive inhibitor, and to the allosteric site, exerting deactivation of the target enzyme. Accordingly, we thought it of interest to test the effect, if any, of orthophosphate on the activity of MptpA. No major effects were detected when 1 mM phosphate was added to reaction mixtures containing *p*-NPP as the substrate (Figure 4C and Table 1). However, the addition of 10 mM phosphate did increase the *K*_m_ for *p*-NPP from 6.24 ± 0.99 to 16.84 ± 1.40 mM (Figure 4C and Table 1). This increase in *K*_m_ implies a *K*_i_ equal to 5.9 mM, a value that is in line with those previously published (2–6 mM) for bovine PTPases.^38−40^

In MptpA, the C(X_5_)R signature of protein tyrosine phosphatases resides in amino acids 11–17, whose sequence is C11-TGNIC-R17. The essential catalytic residues of MptpA (C11, R17, and D126) are located in two tensile regions of the enzyme: (i) at the ends of the P-loop, containing amino acids 12–16, and connecting the β1 sheet and the α1 helix (Figure 1B), and (ii) in the D-loop, containing amino acids 111–131, and connecting β4 and α5 (Figure 1B). MptpA also contains a third loop, the W-loop, the name of which is due to the presence of W48 (Figure 1B). The availability of structural information about the open and closed forms of MptpA^31,32^ was important to show that the D- and W-loop are subjected to a wide movement when the enzyme rearranges its conformation from the open to the closed form, and vice versa (Figure 1C,D). This movement is expected to dramatically affect the environment to which W48 is exposed, with tyrosines Y128 and Y129 much more proximal to W48 in the enzyme closed form (Figure 1D). Therefore, it is reasonable to suppose that the fluorescence intensity of W48 is altered during the open-to-closed conformational rearrangement. To take advantage of this, we decided to construct a synthetic gene, optimized for *E. coli* codon usage and encoding MptpA bearing W48 as the unique enzyme tryptophan. Accordingly, the only other tryptophan of MptpA was substituted with a phenylalanine, introducing into the synthetic gene the site-specific mutation W152F, yielding the nucleotide sequence reported in Figure S1. Using cloning, transformation, overexpression, and purification procedures identical to those applied to wild-type MptpA, we were able to obtain purified MptpA W152F (Figure S3). The availability of MptpA W152F (hereafter named MptpA_sW48_ to indicate the presence of the single tryptophan at site 48) prompted us to investigate the relationship of substrate activation and W-loop movement in MptpA. In particular, we thought it of interest to use MptpA_sW48_ to test the kinetic coupling, if any, between substrate activation and the closure of the enzyme active site, as revealed by tryptophan fluorescence.

As a first test, we decided to perform activity assays using *p*-NPP as the substrate, in the absence or presence of serine, phosphoserine, and orthophosphate (Figures S4 and S5), and to compare the observed catalytic constants with those determined for wild-type MptpA under the same conditions. Overall, no substantial differences were detected between the *k*_cat_ and *K*_m_ values determined for the wild type and for the MptpA_sW48_ enzyme (cf. Table 1 and Tables S1 and S2). Accordingly, we propose that the W152F mutation is not responsible for significant effects on the enzyme catalytic features.

**Figure 5:**
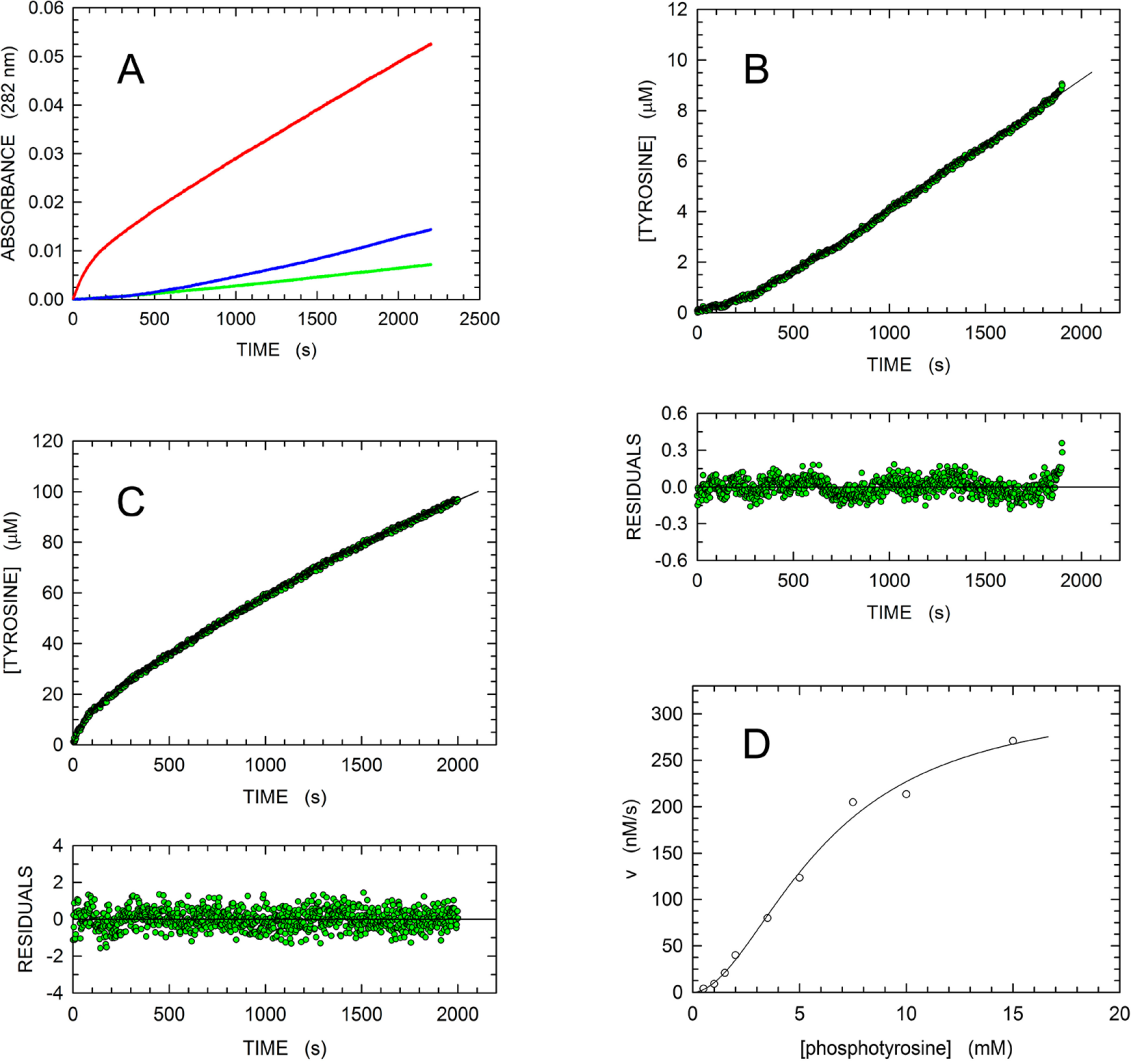
Kinetics of pTyr hydrolysis catalyzed by MptpA_sW48_. (A) Time course of reactions monitored at 282 nm in the presence of 420 nM enzyme and 0.5, 1, or 2.5 mM pTyr (green, blue, and red, respectively). (B) Kinetics of 0.5 mM pTyr hydrolysis catalyzed by 420 nM MptpA_sW48_. To interpret the observed kinetics, a model of substrate activation was used (see Materials and Methods), yielding an integrated equation expressing the concentration of the reaction product as a function of time. The solid line represents the best fit of the model equation to the experimental observations. (C) Kinetics of 5 mM pTyr hydrolysis catalyzed by 420 nM MptpA_sW48_. The solid line represents the best fit of a double-exponential equation to the experimental observations. (D) Dependence of initial reaction velocities on pTyr concentration. The solid line represents the best fit of the Hill equation to the experimental observations.

In addition, we assayed the activity of MptpA_sW48_ at the expense of pTyr, under conditions identical to those used for wild-type MptpA. When the hydrolysis of pTyr was considered, the observed enzyme kinetics was not comparable to that detected in the presence of *p*-NPP (cf. Figure S4A and Figure 5A). In particular, at low pTyr concentrations [e.g., 0.5 and 1 mM (see Figure 5A)], a pronounced lag occurs before the attainment of steady-state conditions. We interpret this lag as being linked to the conversion of MptpA_sW48_ from an inactive to a catalytically competent form, with the inactive form predominant in the absence of a substrate. A very similar kinetic behavior was previously observed with phenylalanine hydroxylase, the enzyme responsible for the conversion of phenylalanine to tyrosine: the reaction product is initially generated at a very slow rate, which continuously increases over 2–5 min to reach a maximal constant value.^41^ To analyze our data, we used a simple model whose assumptions are (i) in the absence of substrate, the enzyme features an inactive conformation (E_i_), (ii) the binding of pTyr to an allosteric site activates MptpA_sW48_, (iii) the pTyr molecule bound to the allosteric site is not subjected to dephosphorylation, and (iv) the reverse reaction (phosphorylation of tyrosine) is negligible. According to this model, we obtained an equation expressing product concentration as a function of time, and containing the *k*_obs_ and *k*′ constants, related to the chemical and to the activation step, respectively (see Materials and Methods).

When this equation was fitted to the data observed in the presence of 0.5 mM pTyr and 420 nM enzyme (Figure 5B), *k*_obs_ and *k*′ were estimated to be equal to 0.1268 ± 0.0002 and (380 ± 4) × 10^–5^ s^–1^, respectively. When the concentration of pTyr in the activity assays was increased >1 mM, the kinetics of substrate dephosphorylation underwent a drastic change (Figure 5A). Instead of the pronounced lag detected in the presence of 0.5–1.0 mM pTyr (Figure 5A,B), when the substrate concentration was increased to 2.5 mM, we observed an initial burst of product, followed by a slower phase of pTyr dephosphorylation (Figure 5A). We interpret this behavior as being due to a fast enzyme activation occurring before the onset of the reaction and to a subsequent inhibition induced by the generation of tyrosine and orthophosphate. It should be noted that an almost identical kinetic behavior was observed with phenylalanine hydroxylase, whose inhibition was observed when the enzyme was preincubated (activated) with phenylalanine.^41^ In addition, it should also be noted that both MptpA and phenylalanine hydroxylase generate tyrosine as the reaction product. We fitted a double-exponential equation to data observed with 420 nM MptpA_sW48_ in the presence of 5 mM pTyr (Figure 5C), yielding *k*_obs_ values equal to (1600 ± 65) × 10^–5^ and (25.4 ± 0.4) × 10^–5^ s^–1^ for the fast and slow phase, respectively (Figure 5C). It should also be noted that the corresponding amplitudes were estimated to be 8.70 ± 0.19 and 219 ± 2 μM for the fast and slow phase, respectively. Therefore, the initial fast phase corresponds to approximately 20 turnovers, ruling out the possibility that the corresponding burst is related to a rate-limiting step (e.g., the regeneration of the free enzyme from the phosphorylated form) occurring after the release of tyrosine by MptpA_sW48_. When a wide range of pTyr concentrations were used to assay MptpA_sW48_ activity, and the initial reaction velocities accordingly detected were expressed as a function of substrate concentration, a sigmoidal behavior was observed, and the Hill equation was therefore fitted to the experimental observations (Figure 5D). From this particular experiment, we estimated *K*_m_ and *k*_cat_ to be equal to 6.12 ± 0.75 mM and 0.76 ± 0.07 s^–1^, respectively, and calculated a Hill coefficient of 1.87 ± 0.22 (Figure 5D). In addition, we used four independent enzyme preparations to evaluate the catalytic constants and determined the mean *K*_m_, *k*_cat_, and Hill coefficient to be equal to 5.12 ± 0.82 mM, 0.53 ± 0.19 s^–1^, and 2.03 ± 0.41, respectively (Table S2). These values were obtained using the initial velocities calculated over the first 20–30 s of reaction time [for assays performed with substrate concentrations yielding a fast and a slow phase, e.g., ≥2.5 mM (Figure 5A,D)]. Remarkably, when we calculated the reaction velocities over 8 min of the slow phase, and we expressed these velocities as a function of substrate concentration, the allosteric nature of MptpA_sW48_ was confirmed, yielding a Hill coefficient of 2.00 ± 0.28 (Figure S6).

The noncatalytic phosphoryl binding site of PTP1B was shown to be competent in binding pTyr, although with a lower affinity when compared to that of the enzyme catalytic site.^28^ To evaluate the *K*_D_ of the pTyr–MptpA_sW48_ complex, we performed surface plasmon resonance (SPR) analyses. This method allows us to determine the dissociation constant and to estimate the stoichiometry of a protein complex in a few seconds and is therefore suitable for MptpA_sW48_ associated with pTyr, as the hydrolysis of this substrate is rather slow (see Figure 5A). The SPR assays revealed an efficient interaction between the immobilized protein and pTyr, as demonstrated by the concentration-dependent responses, and by the clearly discernible exponential curves, during the association and dissociation phases (Figure S7). Intriguingly, the association phase showed a bimodal trend, suggesting a peculiar binding mode. Therefore, we attempted to fit the sensorgrams to single-site (1:1 binding) and double-site (heterogeneous ligand) interaction models. In the first case, a single *K*_D_ of 127 ± 38 nM was calculated, whereas assuming a double-site model, the calculated *K*_D_ values were equal to 315 ± 96 and 2144 ± 915 nM. According to these data, the value of *k*′ [which is equal to *k*_a_[S]/(*K*_D_ + [S])] determined from the reaction kinetics observed in the presence of 0.5 mM pTyr and 420 nM enzyme (Figure 5B) can be considered to represent *k*_a_, the rate constant describing the conversion of MptpA_sW48_ from an inactive to a catalytically competent state. In addition, this is confirmed by superimposing (i) the expected kinetics of MptpA_sW48_ activation, according to a *k*_a_ equal to 0.0038 s^–1^, and (ii) the experimentally determined kinetics of reaction product generation (Figure S8). As the two superimposed kinetics show, enzyme activation is completed when the release of tyrosine reaches a steady state, i.e., approximately 1000 s after reaction started (Figure S8 and Figure 5B). However, it is also important to note that *k*_a_ should be considered a net rate constant,^41^ because the activation of MptpA_sW48_ does most likely consist of a reversible conformational rearrangement. This interpretation of *k*_a_ would explain the lack of a significant lag phase in reaction kinetics observed at the expense of high substrate concentrations [e.g., 5 mM (see Figure 5C)]. The presence of pTyr at concentrations approximately equal to *K*_m_, or higher, would indeed shift the equilibrium toward activation, significantly decreasing the time interval necessary for MptpA_sW48_ activation. This is represented in Figure S8, showing the kinetics of the activation of 420 nM MptpA_sW48_ according to a *k*_a_ equal to 0.038 s^–1^. Under these conditions, 18 s suffices to produce 50% enzyme activation (Figure S8).

To further investigate the sharp transition from a slow to a fast activation of MptpA_sW48_ occurring when the pTyr concentration is increased to values approximately equal to *K*_m_ (Figure 5A–C), we decided to assay enzyme activation in the presence of 0.5 mM pTyr and 10 mM pSer. We have indeed shown that 10 mM pSer is competent in the activation of MptpA_sW48_ when the enzyme catalyzes the hydrolysis of *p*-NPP (Figure S5A). As expected, when the reaction at the expense of 0.5 mM pTyr was assayed in the absence of pSer, a well-defined lag phase was detected before the onset of steady-state kinetics (Figure S9).

Under these conditions, we estimated *k*_obs_ and *k*′ to be equal to 0.0749 ± 0.0002 and 0.0120 ± 0.0003 s^–1^, respectively. Remarkably, when 10 mM pSer was added to the reaction mixture containing 0.5 mM pTyr, the slow phase of enzyme activation was not detected, being replaced by an initial burst of product generation (Figure S9). Fitting a single-exponential equation to these data, we obtained a value for *k*_obs_ equal to (410 ± 9) × 10^–6^ s^–1^ and an amplitude equal to 21.45 ± 0.39 μM (Figure S9). Upon comparison of these values with those determined in the presence of 5 mM pTyr (Figure 5C), it is interesting to note that (i) pTyr is a stronger activator than pSer (yielding a *k*_obs_ that is 40 times higher) and (ii) the two initial phases correspond to approximately the same number of turnovers (i.e., 21 and 26 for the reaction at the expense of 5 mM pTyr and for that observed in the presence of 0.5 mM pTyr and 10 mM pSer, respectively).

**Figure 6:**
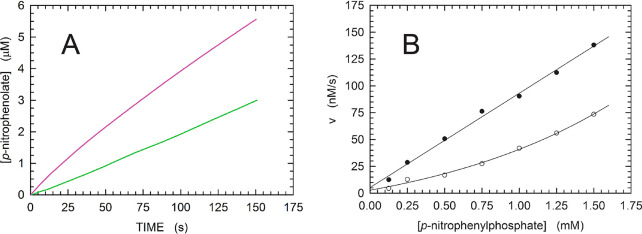
Activation by pSer of *p*-NPP hydrolysis catalyzed by MptpA. (A) Time course of reactions monitored at 405 nm using 210 nM enzyme and 0.5 mM *p*-NPP, in the absence (green) or presence (magenta) of 10 mM pSer. (B) Dependence of initial reaction velocities on the concentration of *p*-NPP in assay mixtures containing 210 nM enzyme, in the absence (○) or presence (●) of 10 mM pSer.

In mechanistic terms, the substrate activation of phenylalanine hydroxylase was interpreted as being related to a conformational transition of the enzyme, as revealed by the competence of phenylalanine in increasing the affinity of the enzyme for phenyl-Sepharose.^42^ For MptpA, it is likely that the conformational transition linked to substrate activation is represented by the closure of the enzyme active site. Moreover, we reasoned that enzyme activation by *p*-NPP might occur significantly faster than the activation triggered by pTyr, making its observation with conventional spectrophotometric assays performed under steady-state conditions difficult. Accordingly, we decided to compare the time course of the reaction catalyzed by MptpA_sW48_ at the expense of 0.5 mM *p*-NPP, in the absence and presence of 10 mM pSer. To facilitate the observation of reaction kinetics, we used in this case an enzyme concentration that was lower than usual, i.e., 210 nM. Interestingly, in the absence of pSer, a lag phase was clearly detected during the initial phase of the reaction, lasting for 20–25 s and preceding the attainment of steady state (Figure 6A), suggesting the occurrence of substrate activation. On the contrary, when 10 mM pSer was added to the reaction mixture, an initial burst of reaction product was observed during the first 80–100 s of reaction time (Figure 6A). To further investigate substrate activation by *p*-NPP, we performed a set of assays using concentrations of this substrate ranging from 0.125 to 1.5 mM. When the observed initial velocities (determined over the first 20 s of reaction time) were represented as a function of *p*-NPP concentration, an exponential behavior was obtained (Figure 6B). However, when reaction mixtures were supplemented with 10 mM pSer, a linear dependence of initial reaction velocity on substrate concentration was detected (Figure 6B). These observations suggest the presence in MptpA of a noncatalytic phosphoryl binding (activating) site, to which, in addition to pTyr and pSer, *p*-NPP can also bind.

**Figure 7:**
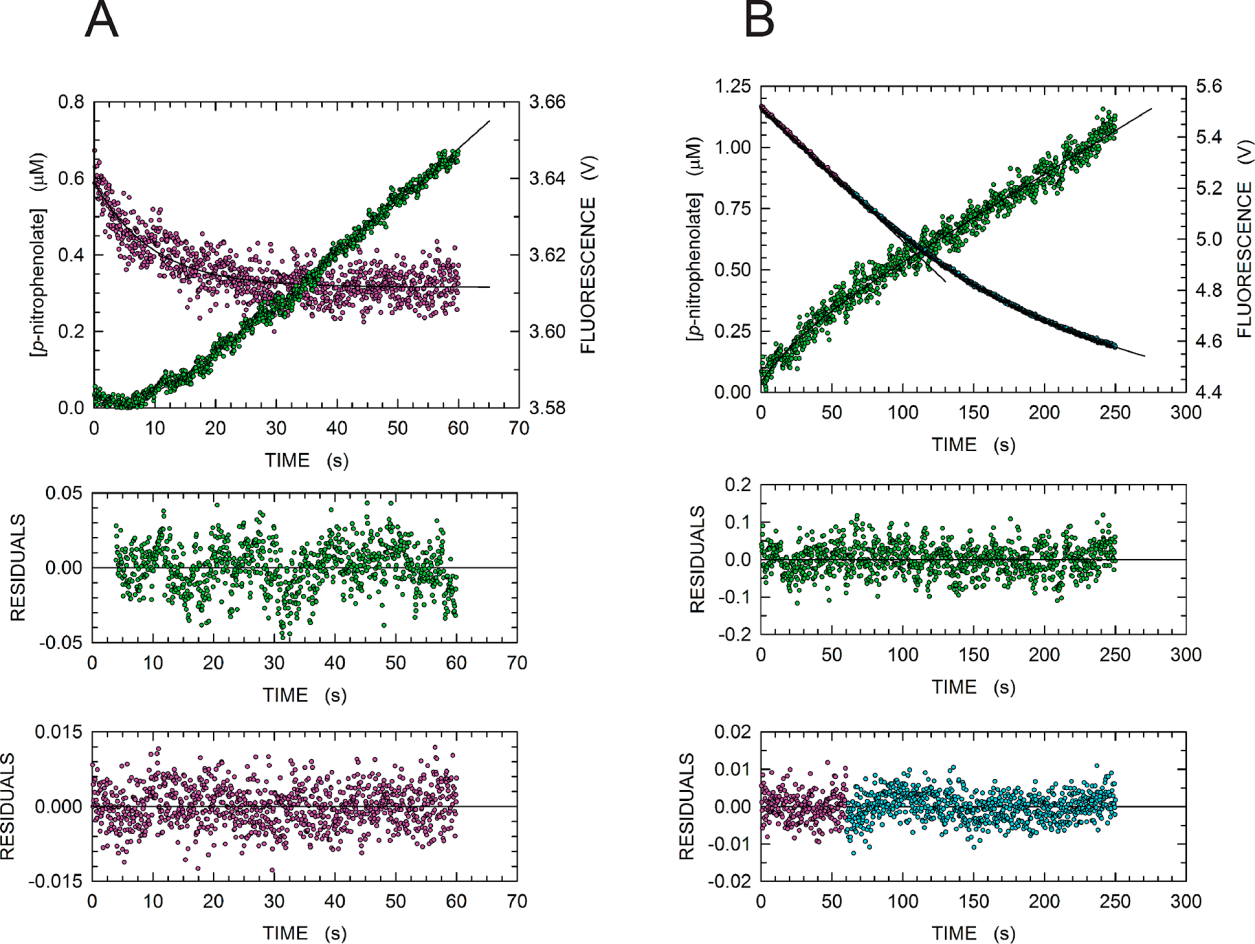
Hydrolysis of *p*-NPP and the open to closed conformational transition of MptpA_sW48_. (A) Time courses of (i) the hydrolysis of 500 μM *p*-NPP catalyzed by 2.25 μM MptpA_sW48_, detected by recording the absorbance at 405 nm (green dots), and (ii) the fluorescence of enzyme W48, as determined by exciting the sample at 280 nm and detecting emission using a long-pass filter (magenta dots). The final concentrations of the substrate and enzyme after mixing the content of the two stopped-flow syringes were 500 and 2.25 μM, respectively. The reaction was assayed at 20 °C in 50 mM Tris-HCl (pH 8) and 2 mM EDTA. The solid line across the green dots represents the best fit of the same equation as in Figure 5B (see Materials and Methods) to the experimental observations. The solid line across the magenta dots represents the best fit of a single-exponential equation to the experimental observations. (B) Time courses of (i) the hydrolysis of 10 μM *p*-NPP catalyzed by 10 μM MptpA_sW48_, detected by recording the absorbance at 405 nm (green dots), and (ii) the fluorescence of enzyme W48, as determined by exciting the sample at 280 nm and detecting emission using a long-pass filter (magenta dots). The final concentrations of the substrate and enzyme after mixing the content of the two stopped-flow syringes were 10 μM. The reaction was assayed at 20 °C in 50 mM Tris (pH 8) and 2 mM EDTA. The solid line across the green dots represents the best fit of the equation *y* = *y*_0_ + *a*[1 – exp(−*bx*)] + *cx* to the experimental observations. The solid lines across the magenta dots represent the best fits of a linear and of a single-exponential equation to the experimental observations.

To inspect the mechanism of substrate activation in MptpA, we performed assays under pre-steady-state conditions (using a stopped-flow method) and observed both the release of *p*-nitrophenolate from *p*-NPP and the fluorescence of MptpA_sW48_ (as previously mentioned, the intensity of W48 fluorescence should be significantly altered by the closure of the enzyme active site). First, when 0.5 mM *p*-NPP was used in the presence of 2.25 μM MptpA_sW48_, substrate activation was observed. Under these conditions, we indeed detected a pronounced lag in the release of *p*-nitrophenolate before the onset of steady-state kinetics (Figure 7A).

Clearly, the time interval of the lag phase was in this case much shorter when compared to that observed when pTyr was used as the substrate (cf. Figures 5B and 7A), yielding *k*_obs_ and *k*′ values of 0.0543 ± 0.0003 and 0.085 ± 0.003 s^–1^, respectively. We propose that for the reaction at the expense of *p*-NPP *k*′ also approximately represents *k*_a_, as was determined for pTyr. Remarkably, the release of *p*-nitrophenolate did reach a steady-state regime when the observed fluorescence decrease of W48 reached completion (Figure 7A). This decrease is satisfactorily interpreted by a single-exponential equation with a *k*_obs_ equal to 0.106 ± 0.005 s^–1^ (Figure 7A). The experiments performed in the presence of 0.5 mM substrate [pTyr or *p*-NPP (Figures 5B and 7A)] indicate that the rate constants related to product release are on the same order of magnitude (0.1268 and 0.0543 s^–1^ for pTyr and *p*-NPP, respectively) and that the two activation rate constants markedly differ, being 0.0038 and 0.085 s^–1^ for pTyr and *p*-NPP, respectively. It is also remarkable that the activation rate constant determined for *p*-NPP (0.085 s^–1^) is rather similar to the *k*_obs_ observed for enzyme closure (0.106 s^–1^), suggesting a link between activation and competence in poising active site closure.

To ascertain whether free and inactive MptpA_sW48_ is in equilibrium with a free and active form, we performed a single-turnover experiment, in the presence of equimolar concentrations of *p*-NPP and the enzyme. In particular, we assayed under pre-steady-state conditions both *p*-nitrophenolate release (at 405 nm) and MptpA_sW48_ tryptophan fluorescence, after mixing using our stopped-flow 20 μM enzyme with 20 μM *p*-NPP. Surprisingly, a tiny, albeit significant, initial fast phase of *p*-nitrophenolate generation was detected (Figure 7B). This initial burst was followed by a slow phase, and the observed kinetics was interpreted by fitting the equation *y* = *y*_0_ + *a*[1 – exp(−*bx*)] + *cx* to the observed data (with *y*_0_ and *x* indicating the intercept at *t*_0_ and time, respectively). By this means, we estimated the amplitude and the first-order rate constant of the initial burst to be equal to 0.137 ± 0.009 μM and 0.033 ± 0.005 s^–1^, respectively (Figure 7B). In addition, we calculated the *k*_obs_ of the slow phase to be equal to (400.0 ± 4.2) × 10^–5^ μM/s (Figure 7B). The decrease in tryptophan fluorescence obeyed a complex kinetics, composed of an initial linear phase, lasting for the first 60 s, followed by an exponential decrease (Figure 7B). The linear and exponential phase can be satisfactorily accounted for by a rate equal to 5.00 ± 0.01 mV/s and by a rate constant equal to (6.00 ± 0.03) × 10^–3^ s^–1^ (Figure 7B). According to the results of the single-turnover experiment, we propose that a small fraction, approximately equal to 1–2% of free MptpA_sW48_, is in the form of active enzyme and does not require substrate activation to exert catalysis.

**Figure 8:**
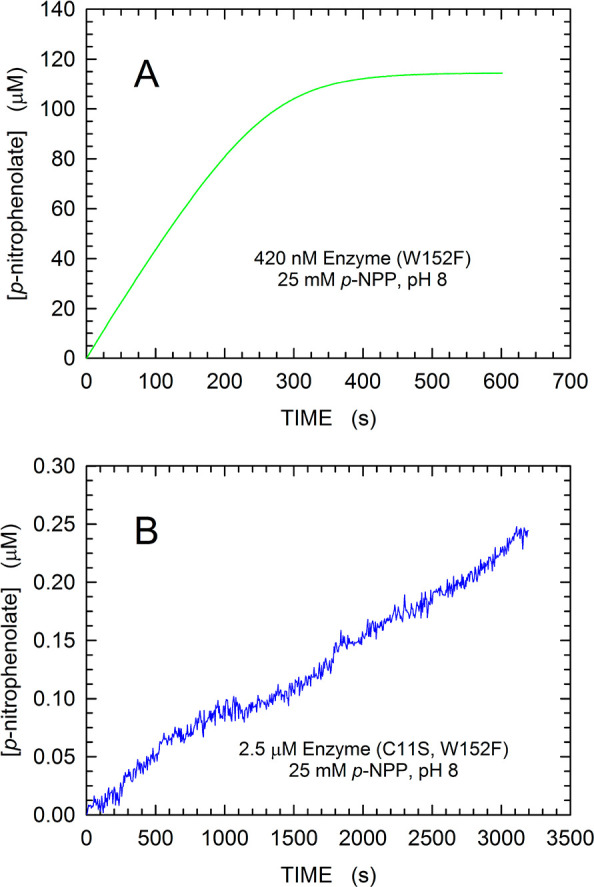
Hydrolysis of *p*-NPP by MptpA_sW48_ and by MptpA double mutant C11S W152F. (A) Kinetics of *p*-nitrophenolate generation by 420 nM MptpA_sW48_ at the expense of 25 mM *p*-NPP [50 mM Tris-HCl and 2 mM EDTA (pH 8)]. (B) Activity of 2.5 μM MptpA double mutant C11S W152F observed under the same assay conditions used for MptpA_sW48_.

Finally, we reasoned that the binding and the action of MptpA inhibitors could be conveniently assayed by monitoring the fluorescence changes of W48 triggered by the closure of the enzyme active site. In particular, to provide a useful tool for assaying the association of inhibitors with MptpA, we introduced into MptpA_sW48_ the C11S site-specific mutation (Figure S1), and we overexpressed and purified this double mutant according to the same procedures previously used for the wild type and MptpA_sW48_. Remarkably, the site-specific C11S substitution was found to be responsible for an almost complete lack of activity at the expense of *p*-NPP. We were indeed unable to observe a consistent hydrolysis of *p*-NPP even in the presence of a high concentration (2.5 μM) of the C11S W152F double mutant (Figure 8). In quantitative terms, we observed activities with 420 nM MptpA_sW48_ and 2.5 μM C11S W152F MptpA equal to 451.7 ± 0.7 nM/s and 70.6 ± 0.3 pM/s, respectively. Therefore, the binding of a phosphorylated compound to MptpA C11S W152F can be tested over a quite long time interval (e.g., hours) without any concomitant dephosphorylation, and the fluorescence of the unique tryptophan of the inactive enzyme can be used as a probe of the conformational rearrangements triggered by the association event. Moreover, the comparison of the fluorescence changes occurring in MptpA C11S W152F with those taking place in MptpA_sW48_ could conveniently discriminate between (i) phosphorylated compounds targeting the active site, therefore acting competitively, and (ii) phosphorylated ligands of the noncatalytic site, triggering activation or noncompetitive inhibition. By this means, the identification of allosteric inhibitors inducing the active site to idle in the closed or open conformation would be of particular interest, therefore preventing the conformational transitions essential for performing multiple catalytic cycles.

## Conclusions

We report here on substrate activation of the low-molecular weight tyrosine phosphatase from *M. tuberculosis* (MptpA). Both substrates tested, *p*-NPP and pTyr, were effective in activating the enzyme, as revealed by the increase in reaction velocity detected when activity assays were performed using low substrate concentrations. The activation of MptpA most likely occurs via a reversible conformational rearrangement of the enzyme, leading to a catalytically competent form. In mechanistic terms, MptpA is activated through the action of an allosteric site, where the binding of a substrate molecule not subjected to hydrolysis occurs. Alternatively, phosphoserine, which is not a substrate for MptpA, can activate the enzyme by binding the allosteric site. The MptpA allosteric site features a high affinity for the binding of pTyr, with the resulting complex characterized by a submicromolar *K*_D_. Considering this high affinity, it is our hope that the observations reported here will trigger the search of antituberculosis drugs that can target the allosteric site of MptpA and exert a specific inhibitory action. Toward this end, unraveling the structural determinants of allosteric MptpA ligands responsible for enzyme activation or inhibition will represent a major challenge.

## Supporting Information Available

Sequence of the synthetic MptpA, MptpA W152F, and MptpA W152F C11S genes (Figure S1), MptpA expression and purification (Figure S2), MptpA_sW48_ expression and purification (Figure S3), dependence of MptpA_sW48_-catalyzed reaction velocities on *p*-NPP concentration (Figure S4), activation by pSer and inhibition by orthophosphate of MptpA_sW48_ (Figure S5), values of *K*_m_, *V*_max_, and *k*_cat_ determined for MptpA W152F using *p*-NPP as the substrate (Table S1), values of *K*_m_, *V*_max_, and *k*_cat_ determined for MptpA W152F using *p*-NPP or pTyr as the substrate (Table S2), dependence of MptpA_sW48_-catalyzed reaction velocities on pTyr concentration (Figure S6), surface plasmon resonance assays (Figure S7), kinetics of MptpA_sW48_ activation (Figure S8), and activation of pTyr hydrolysis catalyzed by MptpA_sW48_ (Figure S9) (PDF)

## Accession Codes

The UniProt accession code for the low-molecular weight protein tyrosine phosphatase from *M. tuberculosis* (MptpA) is P9WIA1.
