# Supplementary material for: Substrate Activation of the Low-Molecular Weight Protein Tyrosine Phosphatase from Mycobacterium tuberculosis
Source: Biochemistry. 2020 Mar 6;59(11):1137–48. doi: 10.1021/acs.biochem.0c00059 (PMC7997110; doi:10.1021/acs.biochem.0c00059)
Supplement: Supplementary file 1 — bi0c00059_si_001.pdf [file bi0c00059_si_001.pdf]

Supporting Information for:

Substrate activation of the low-molecular-weight protein tyrosine phosphatase from *Mycobacterium tuberculosis*

Alessandra Stefan,[†,#] Fabrizio Dal Piaz,[‡] Antonio Girella,[†] and Alejandro Hochkoeppler[†,#,*]

[†] Department of Pharmacy and Biotechnology, University of Bologna, 40136, Bologna, Italy

[#] CSGI, University of Firenze, 50019 Sesto Fiorentino (Firenze), Italy

[‡] Department of Medicine, University of Salerno, 84084 Fisciano (Salerno), Italy

Corresponding Author

* E-mail: a.hochkoeppler@unibo.it

Contents

Figure S1. Sequence of the synthetic MptpA, MptpA W152F and MptpA W152F-C11S genes

Figure S2. MptpA expression and purification

Figure S3. $MptpA_{sW48}$ expression and purification

Figure S4. Dependence of $MptpA_{sW48}$-catalyzed reaction velocities on *p*-NPP concentration

Figure S5. Activation by pSer and inhibition by orthophosphate of $MptpA_{sW48}$

Table S1. Values of K_m, V_{max}, and k_{cat} determined for MptpA W152F using *p*-NPP as substrate

Table S2. Values of K_m, V_{max}, and k_{cat} determined for MptpA W152F using *p*-NPP or pTyr as substrate

Figure S6. Dependence of $MptpA_{sW48}$-catalyzed reaction velocities on pTyr concentration

Figure S7. Surface Plasmon Resonance assays

Figure S8. Kinetics of $MptpA_{sW48}$ activation

Figure S9. Activation of pTyr hydrolysis catalysed by $MptpA_{sW48}$

Figure S1

A

CCATGGGATCAGATCCCCTACACGTAACATTCGTTTGTACTGGGAATATATGTAGGAGCCCGATGG
CGGAGAAGATGTTTGCGCAGCAACTGCGTCACCGTGGTCTGGGTGATGCGGTGCGTGTTACCAGCG
CGGGCACCGGTAACTGGCATGTTGGTAGCTGCGCGGATGAGCGTGCGGCGGGTGTTCTGCGTGCGC
ACGGCTACCCGACCGATCACCGTGCGGCGCAAGTGGGCACCGAACATCTGGCGGCGGACCTGCTGG
TTGCGCTGGATCGTAACCACGCGCGTCTGCTGCGTCAACTGGGCGTGGAGGCGGCGCGTGTTCGTA
TGCTGCGTAGCTTTGACCCGCGTAGCGGCACCCATGCGCTGGACGTGGAAGATCCGTACTATGGCG
ACCACAGCGATTTCGAGGAAGTGTTTGCGGTTATTGAGAGCGCGCTGCCGGGTCTGCACGACTGGG
TTGATGAACGTCTGGCGCGTAACGGCCCGAGCTAACTGCAG

B

CCATGGGATCAGATCCCCTACACGTAACATTCGTTTGTACTGGGAATATATGTAGGAGCCCGATGG
CGGAGAAGATGTTTGCGCAGCAACTGCGTCACCGTGGTCTGGGTGATGCGGTGCGTGTTACCAGCG
CGGGCACCGGTAACTGGCATGTTGGTAGCTGCGCGGATGAGCGTGCGGCGGGTGTTCTGCGTGCGC
ACGGCTACCCGACCGATCACCGTGCGGCGCAAGTGGGCACCGAACATCTGGCGGCGGACCTGCTGG
TTGCGCTGGATCGTAACCACGCGCGTCTGCTGCGTCAACTGGGCGTGGAGGCGGCGCGTGTTCGTA
TGCTGCGTAGCTTTGACCCGCGTAGCGGCACCCATGCGCTGGACGTGGAAGATCCGTACTATGGCG
ACCACAGCGATTTCGAGGAAGTGTTTGCGGTTATTGAGAGCGCGCTGCCGGGTCTGCACGAC**TTT**G
TTGATGAACGTCTGGCGCGTAACGGCCCGAGCTAACTGCAG

C

CCATGGGATCAGATCCCCTACACGTAACATTCGTT**TCT**ACTGGGAATATATGTAGGAGCCCGATGG
CGGAGAAGATGTTTGCGCAGCAACTGCGTCACCGTGGTCTGGGTGATGCGGTGCGTGTTACCAGCG
CGGGCACCGGTAACTGGCATGTTGGTAGCTGCGCGGATGAGCGTGCGGCGGGTGTTCTGCGTGCGC
ACGGCTACCCGACCGATCACCGTGCGGCGCAAGTGGGCACCGAACATCTGGCGGCGGACCTGCTGG
TTGCGCTGGATCGTAACCACGCGCGTCTGCTGCGTCAACTGGGCGTGGAGGCGGCGCGTGTTCGTA
TGCTGCGTAGCTTTGACCCGCGTAGCGGCACCCATGCGCTGGACGTGGAAGATCCGTACTATGGCG
ACCACAGCGATTTCGAGGAAGTGTTTGCGGTTATTGAGAGCGCGCTGCCGGGTCTGCACGAC**TTT**G
TTGATGAACGTCTGGCGCGTAACGGCCCGAGCTAACTGCAG

Figure S1. **Sequence of the synthetic MptpA, MptpA W152F and MptpA W152F-C11S genes.** Each gene was cloned into the pET-Duet-1 vector using *Nco*I and *Pst*I restriction sites (underlined). (A) Nucleotide sequence of MptpA wild-type. (B) Nucleotide sequence of MptpA W152F: the mutation W152F was introduced by converting the tryptophan codon TGG to a phenylalanine codon TTT (in bold). (C) Nucleotide sequence of MptpA W152F-C11S: in addition to the W152F mutation, a second mutation was introduced to convert the cysteine codon TGT to a serine codon TCT (in bold).

Figure S2

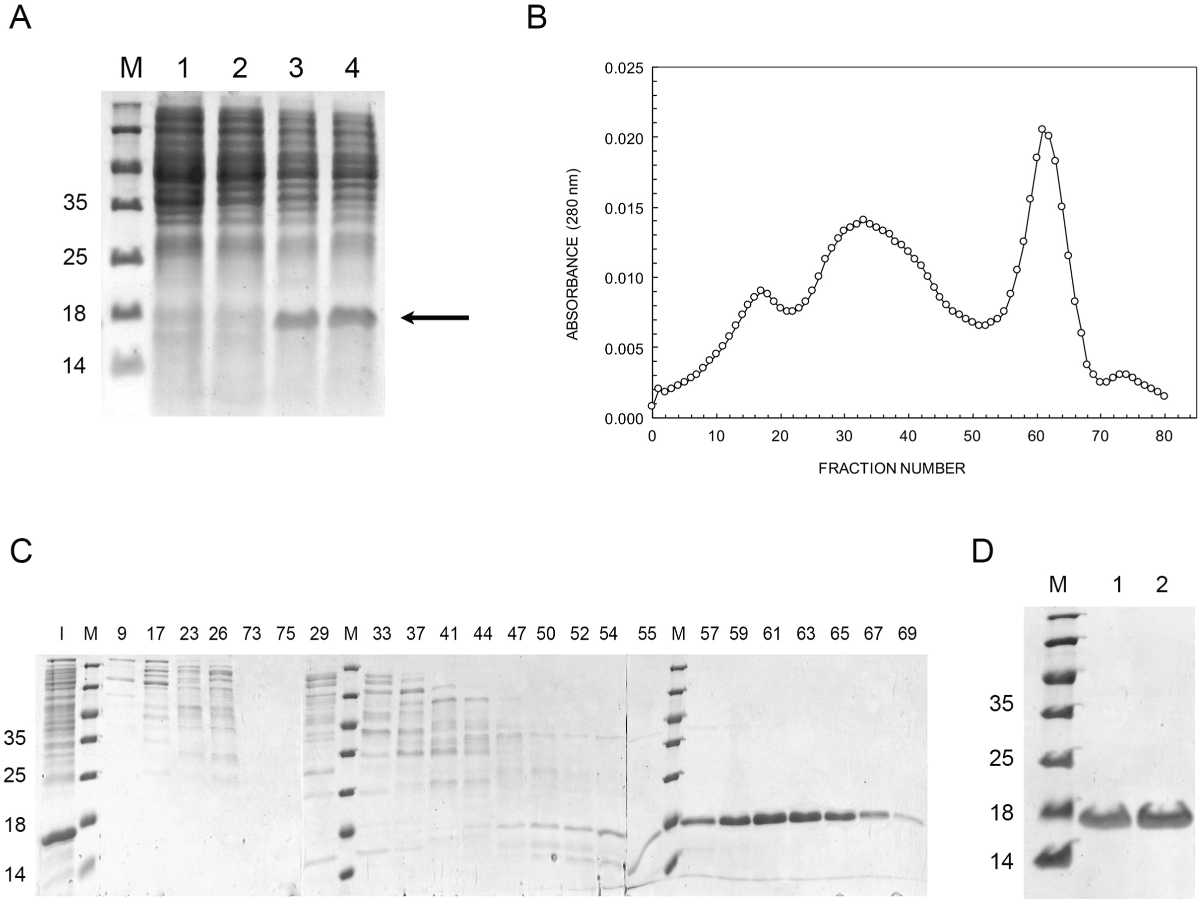

Figure S2. **MptpA expression and purification.** (A) SDS-PAGE (15% polyacrylamide) of total and soluble protein fractions obtained from *E. coli* BL21(DE3)/pETDuet1-MptpA not-induced or induced cultures (lanes 1-2 and 3-4, respectively); lane M: molecular mass markers. The molecular masses of standards are indicated at the left. The arrow indicates the band corresponding to recombinant MptpA (approximately 17 kDa). (B) Elution profile of the gel filtration chromatography (Superdex 200 column) performed to purify MptpA; (C) SDS-PAGE (15% polyacrylamide) of fractions eluted from the gel filtration column; lane M: molecular mass markers; I: input. Fraction numbers are indicated at the top, and the molecular masses of standards are indicated at the left. (D) SDS-PAGE (15% polyacrylamide) of the purified MptpA and MptpA$_{sW48}$ proteins (lane 1 and 2, respectively). Approximately 4 μg of protein were run per lane. M: molecular mass markers. The molecular masses of standards are indicated at the left.

Figure S3

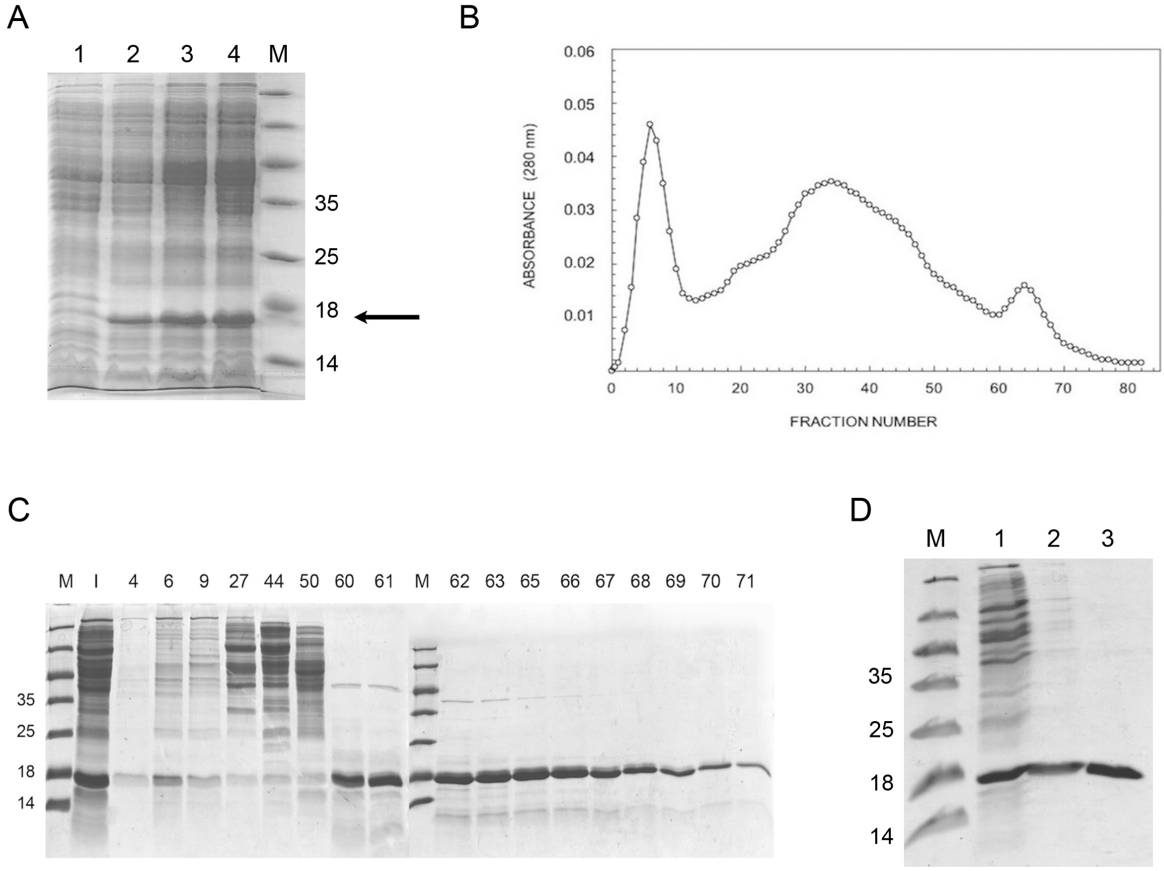

Figure S3. MptpA$_{sW48}$ expression and purification. (A) SDS-PAGE (15% polyacrylamide) of total proteins extracted from *E. coli* BL21(DE3)/pETDuet1-MptpA(W152F) not-induced or induced cultures (lanes 1 and 2, respectively); lanes 3 and 4: total and soluble protein fractions obtained from induced cultures, respectively; lane M: molecular mass markers. The molecular masses of standards are indicated at the right. The arrow indicates the band corresponding to recombinant MptpA$_{sW48}$ (approximately 17 kDa). (B) Elution profile of the gel filtration chromatography (Superdex 200 column) performed to purify MptpA$_{sW48}$; (C) SDS-PAGE (15% polyacrylamide) of fractions eluted from the gel filtration column; lane M: molecular mass markers; I: input. Fraction numbers are indicated at the top, and the molecular masses of standards are indicated at the left. (D) SDS-PAGE analysis of MptpA$_{sW48}$ samples isolated along the purification steps: after phenyl-sepharose chromatography (lane 1), after gel filtration chromatography (lane 2), pooled concentrated fractions after gel filtration (lane 3). M: molecular mass markers. The molecular masses of standards are indicated at the left.

Figure S4

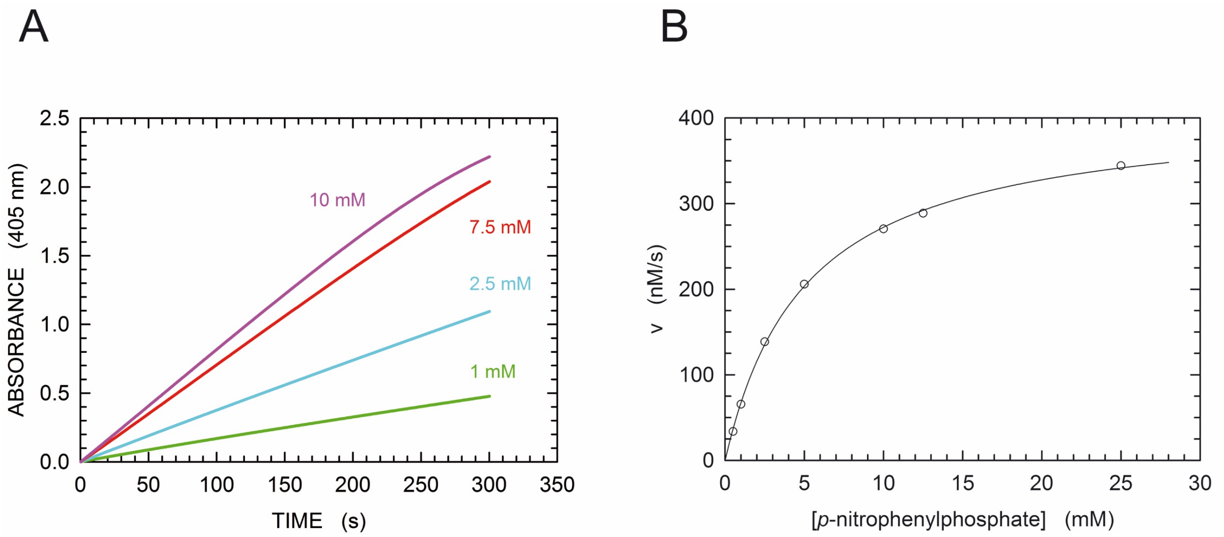

Figure S4. Kinetics of *p*-NPP hydrolysis catalysed by MptpA$_{\mathbf{sW48}}$. (A) Time-course of reactions monitored at 405 nm in the presence of 420 nM enzyme and *p*-NPP at the indicated concentrations. (B) Dependence of initial reaction velocities on *p*-NPP concentration. The continuous line represents the best fit of the Michaelis-Menten equation to the experimental observations.

Figure S5

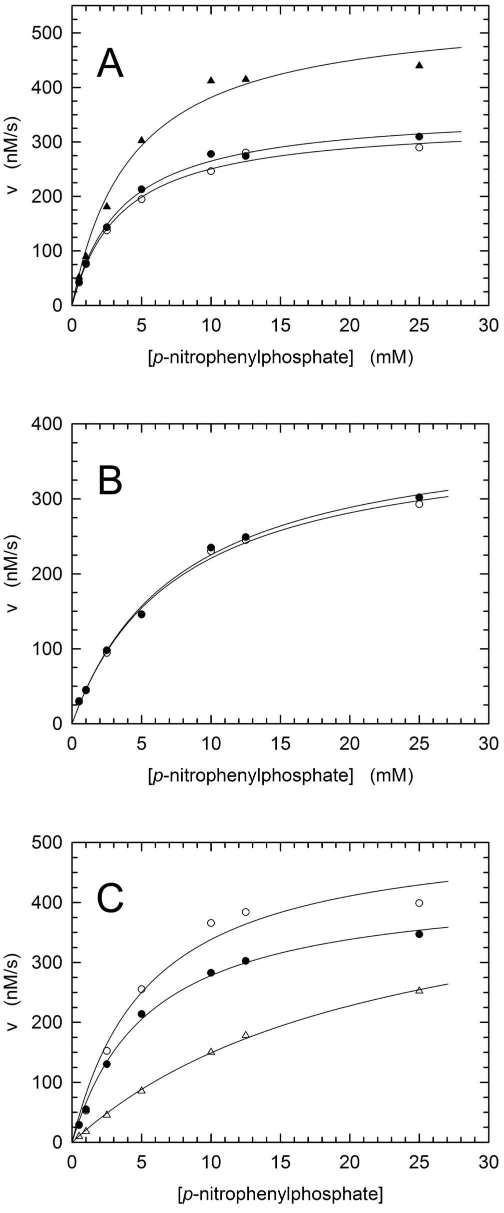

Figure S5. Activation and inhibition of MptpA$_{sW48}$. (A) Initial velocities of *p*-NPP hydrolysis as a function of substrate concentration, assayed in the absence (open circles), or in the presence of 1 mM (filled circles) or 10 mM (filled triangles) phosphoserine. (B) Initial velocities of *p*-NPP hydrolysis as a function of substrate concentration, assayed in the absence (open circles), or in the presence of 10 mM (filled circles) serine. (C) Initial velocities of *p*-NPP hydrolysis as a function of substrate concentration, assayed in the absence (open circles), or in the presence of 1 mM (filled circles) or 10 mM (open triangles) orthophosphate.

Table S1

Table S1. Values of K_m, V_{max}, and k_{cat} determined for MptpA W152F using *p*-NPP as substrate. The effect on catalytic constants induced by the addition to reaction mixtures of phosphoserine, serine or orthophosphate, at the indicated concentrations, is reported.

	K_m (mM)	V_{max} (nM/s)	k_{cat} (s^{-1})
***p*-NPP**	3.5 ± 0.4	339 ± 11	0.81 ± 0.03
***p*-NPP and 1 mM pSer**	3.6 ± 0.3	360 ± 9	0.86 ± 0.02
***p*-NPP and 10 mM pSer**	4.3 ± 0.7	546 ± 31	1.30 ± 0.07
***p*-NPP**	7.6 ± 0.7	388 ± 15	0.92 ± 0.04
***p*-NPP and 10 mM Ser**	7.8 ± 0.8	401 ± 16	0.95 ± 0.04
***p*-NPP**	5.5 ± 1.2	523 ± 43	1.25 ± 0.10
***p*-NPP and 1 mM P_i**	5.5 ± 0.5	432 ± 14	1.03 ± 0.03
***p*-NPP and 10 mM P_i**	22.3 ± 1.7	482 ± 22	1.15 ± 0.05

Table S2

Table S2. Values of K_m, V_{max}, and k_{cat} determined for MptpA W152F using *p*-NPP or pTyr as substrate. Four batches of enzyme, independently purified, were used to assess the catalytic constants. The final concentration of enzyme in the activity assays performed with each batch is reported in brackets.

	K_m (mM)	V_{max} (nM/s)	kcat (s^{-1})	Mean K_m	Mean k_{cat}
***p*-NPP**	5.2	585 (640 nM E)	0.91	4.33 ± 0.95	0.85 ± 0.12
	3.5	339 (420 nM E)	0.81		
	5.1	412 (420 nM E)	0.98		
	3.5	296 (420 nM E)	0.70		
**pTyr**	5.2	312 (640 nM E)	0.49	5.12 ± 0.82	0.53 ± 0.19
	4.1	126 (420 E)	0.30		
	5.1	242 (420 E)	0.58		
	6.1	318 (420 E)	0.76		
**Hill coefficient**	2.3	**Mean Hill coefficient**	2.03 ± 0.41		
	2.4				
	1.5				
	1.9				

Figure S6

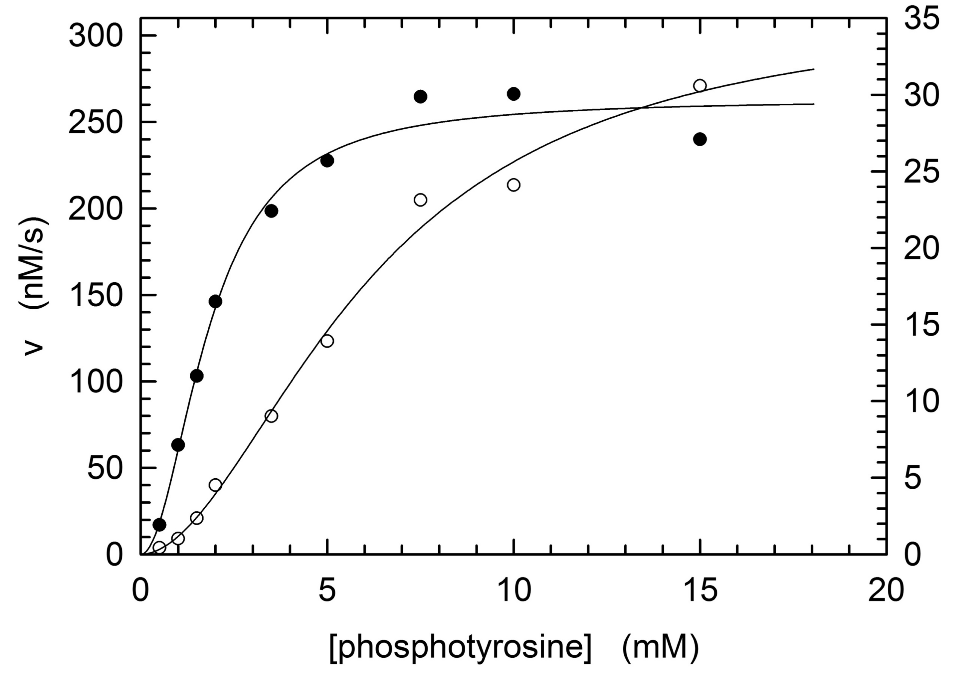

Figure S6. Dependence of reaction velocities on pTyr concentration. The MptpA$_{sW48}$ activity at the expense of pTyr was determined under steady-state conditions (same enzyme and substrate concentrations as in Figure 6D). Reaction velocities were calculated by fitting a straight-line equation to the data observed over a time interval of 20-30 seconds of the fast phase (empty circles) or over 8 minutes of the slow phase (filled circles) of the observed kinetics. The continuous lines represent the best fit of the Hill equation to the experimental observations.

Figure S7

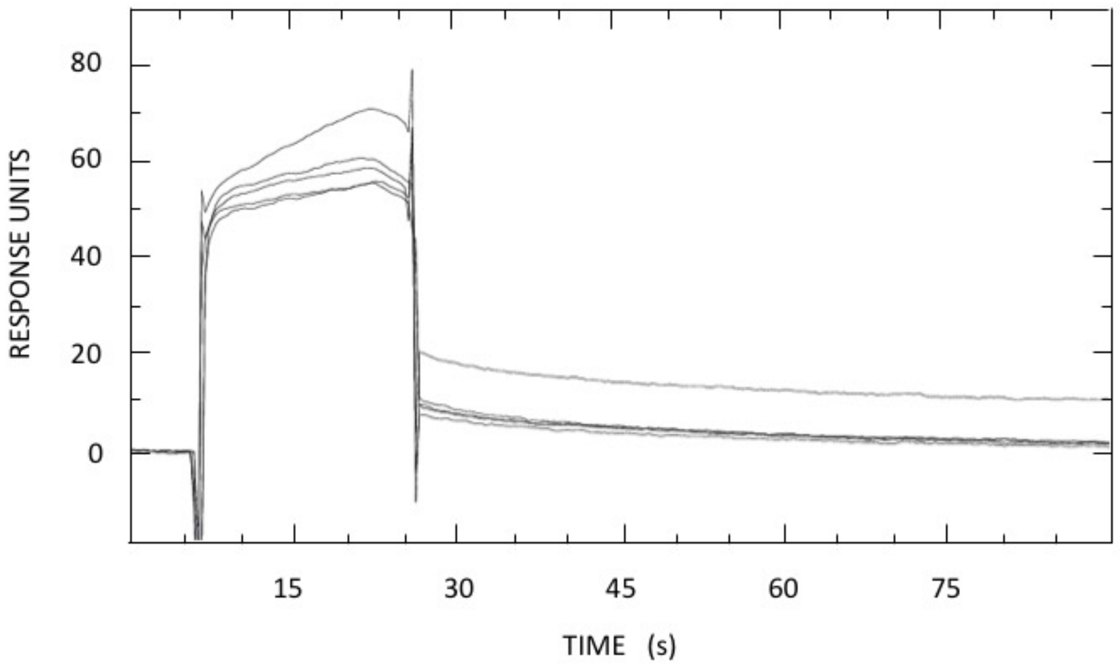

Figure S7. **Surface Plasmon Resonance assays of pTyr binding to MptpA$_{sW48}$.** Sensorgrams obtained by injecting different concentrations (from 0.025 nM to 1 μM) of pTyr on a MptpA$_{sW48}$ modified sensor chip.

Figure S8

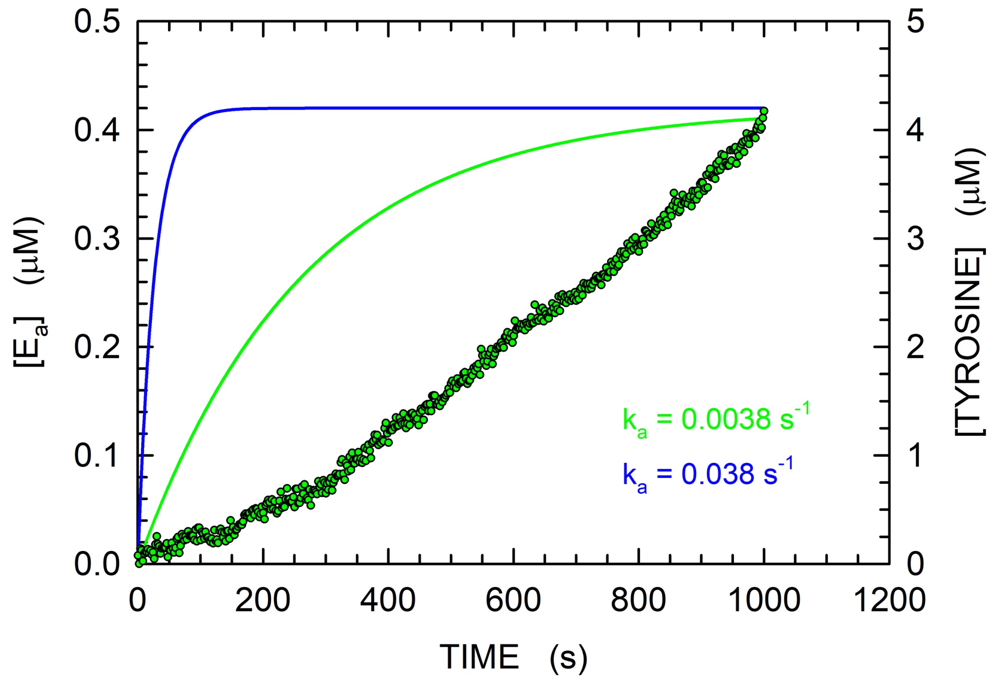

Figure S8. **Kinetics of MptpA$_{sW48}$ activation.** The expected time-course of MptpA$_{sW48}$ (420 nM) activation according to a k_a equal to 0.0038 s^{-1} is reported with a green line. The green dots represent the observed generation of Tyrosine, catalysed by 420 nM MptpA$_{sW48}$ in the presence of 0.5 mM pTyr as substrate (these data are the same shown in Figure 6B). For comparison, the expected kinetics of MptpA$_{sW48}$ (420 nM) activation according to a k_a equal to 0.038 s^{-1} is reported with a blue line.

Figure S9

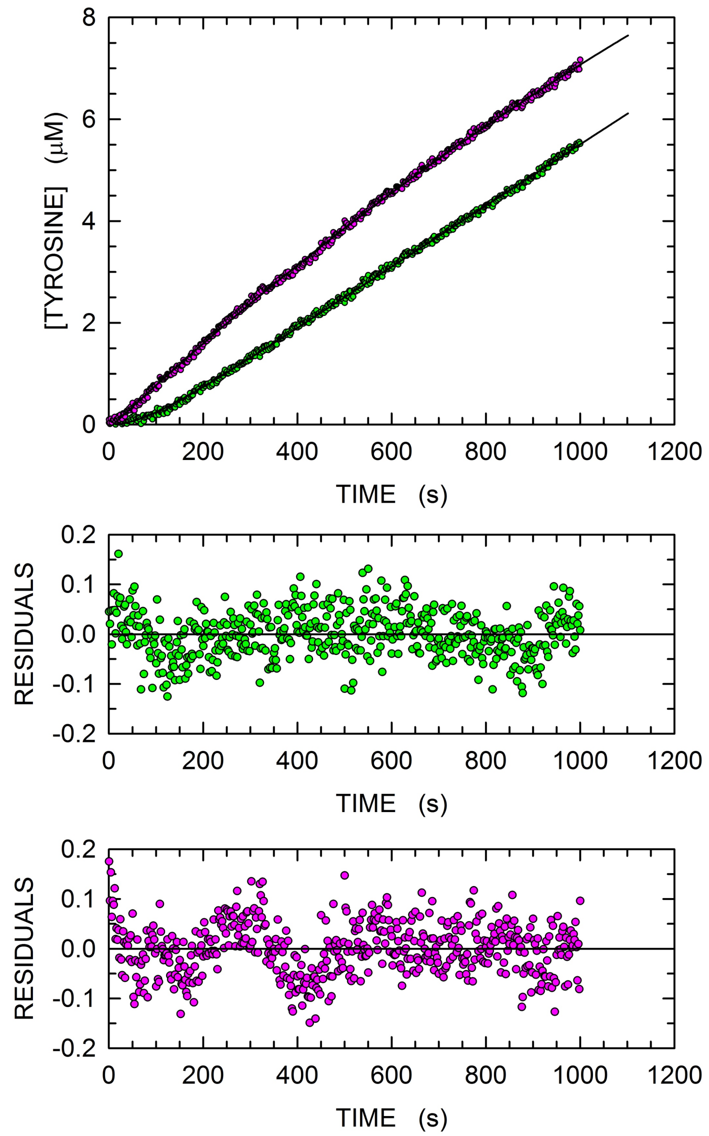

Figure S9. Activation by pSer of pTyr hydrolysis catalysed by MptpA$_{sW48}$. Time-course of reactions monitored at 282 nm in the presence of 840 nM enzyme and 0.5 mM pTyr in the absence (green dots) or in the presence (magenta dots) of 10 mM pSer. Both reactions were assayed in 50 mM Tris-HCl, 2 mM EDTA, pH 8. The continuous lines represent: i) the best fit of the equation modelling substrate activation (see Methods) to the data obtained in the absence of pSer (green dots); ii) the best fit of a single exponential equation to the experimental observations (magenta dots) observed in the presence of 10 mM pSer.
